# Novel uses of thyroid hormones in cardiovascular conditions

**DOI:** 10.1007/s12020-019-02050-4

**Published:** 2019-10-15

**Authors:** Salman Razvi

**Affiliations:** Institute of Genetic Medicine and Queen Elizabeth Hospital, Newcastle University, Centre for Life, Central Park, Newcastle upon Tyne, NE1 3BZ UK

**Keywords:** Thyroid hormones, Acute myocardial infarction, Heart failure, Coronary artery bypass grafting, Cardiac transplantation, Left ventricular function

## Abstract

Thyroid hormone levels are reduced in cardiovascular diseases and this phenomenon is associated with worse outcomes. It is unclear whether the changes in thyroid hormone bioavailability to the affected myocardium are beneficial or if this is a maladaptive response. Experimental studies from animal models of acute myocardial infarction (AMI) suggest that thyroid hormone treatment may be beneficial. There is limited data available on the use of thyroid hormones in patients with AMI and heart failure and this suggests that treatment to normalise thyroid hormone levels may be safe and potentially efficacious. Similarly, evidence of thyroid hormone therapy in patients undergoing cardiac surgery or during cardiac transplantation is limited. It is therefore difficult to draw any firm conclusions about benefits or risks of thyroid hormone treatment in these conditions. Large scale clinical trials of thyroid hormones in patients with cardiac conditions are required to confirm safety and evaluate efficacy. Furthermore, it needs to be elucidated which hormone to administer (thyroxine or triiodothyronine), when in the disease pathway to treat, dose of thyroid hormone to administer, and which parameters to utilise to assess safety and efficacy. Until these important questions are answered thyroid hormone therapy in cardiovascular diseases must remain within the research domain.

## Introduction

Over the course of the last few decades changes in lifestyle have led to increases in atherosclerotic conditions, such as obesity, hypertension, and type 2 diabetes mellitus. These lifestyle-related morbidities together with an increased lifespan has caused a steep rise in the incidence and prevalence of cardiovascular diseases (CVD). CVD, of which ischaemic heart disease is the predominant condition, affects hundreds of millions of individuals across the world [1]. Behavioural changes, therapeutic targeting of CVD risk factors, and improvements in the management of acute coronary events have slowed the increase in CVD overall but has led to increases in chronic health conditions and health-related spending [2]. For instance, the reduction in short-term mortality after acute coronary events has resulted in an increased burden of longer-term sequelae such as heart failure. Newer therapies are required to alleviate the problem of CVD on a global scale in a cost-effective manner.

**Table 1:** Effects of thyroid hormone T3 on various cardiovascular and neuroendocrine parameters in patients with heart failure

Parameter and effect
Left ventricular systolic function ⇑
Left ventricular diastolic function ⇑
Systemic vascular resistance ⇓
Noradrenaline ⇓
Aldosterone ⇓
Natriuretic peptide synthesis ⇑
Myocardial blood flow ⇑

Thyroid hormones regulate development and metabolism of various organs with the cardiovascular system being a major target for its actions [3]. Minor changes in thyroid hormones, even within the reference range, are associated with increased risk of CVD and cardiovascular risk factors [4]. Thus, it is possible that modulating thyroid hormone levels could be utilised as a therapeutic option to improve health. In addition, illness itself can lead to reversible changes in thyroid function—a process termed as non-thyroidal illness (NTI). The perturbations in thyroid function in NTI are not directly attributed to primary hypothalamic, pituitary, or thyroid diseases [5]. It has been debated for some time whether the changes in thyroid function observed in NTI are protective as they minimise protein catabolism, or whether they represent pathological thyroid hormone deficiency that could potentially benefit from hormone replacement therapy. The argument centres around the crucial effects of thyroid hormones on processes involved in repair mechanisms. Thyroid hormone deficiency could lead to a number of cardiovascular effects including decreased cardiac output, increased systemic vascular resistance, or delayed repair from injury, and immune dysfunction [6]. Unnecessary thyroid hormone replacement, on the other hand, increases oxygen demand and cardiac work and induces tachyarrhythmias, particularly atrial fibrillation, coronary spasm, and cardiac ischaemia even in the absence of significant coronary artery disease [5]. As the heart and vascular system are important target organs of thyroid hormone action this review will focus on novel uses of thyroid hormone in cardiovascular conditions.

## Historical perspective on the use of thyroid hormones in CVD

Thyroid extract was the first hormonal preparation introduced into clinical medicine more than a century ago. In 1891, George Murray, a pathologist in Newcastle, England, injected sheep thyroid extract subcutaneously to a 46-year-old woman with typical clinical features of myxoedema [7]. The clinical effect was swift and very effective, and the patient went on to live for almost 30 years on thyroid hormone substitution therapy and eventually died of heart failure [8]. Edward Kendall crystallised thyroxine (T4) in 1914 and Harington and Barger identified its chemical structure and synthesised it in the laboratory in 1927 [9]. Subsequently, Pitt-Rivers and Gross detected the second thyroid hormone triiodothyronine (T3) [10]. Treatment with injectable and then oral thyroid extract remained the mainstay of treatment until the 1950s probably due to the high cost of the synthetic levothyroxine [11]. Later, as levothyroxine became more widely available at reasonable cost the use of thyroid extract became virtually non-existent. In addition, there have been concerns about the ratio of T3:T4 in the porcine-derived thyroid extract which is much higher than the amount secreted by the human thyroid gland [12].

When the striking effects of thyroid hormones on serum cholesterol levels became apparent, cholesterol determination and measurement of basal metabolic rate were utilised as indirect diagnostic tests of thyroid function, particularly in the absence of specific hormonal assays of thyroid function [9]. Furthermore, atherosclerosis of peripheral arteries was found to be associated with low protein-bound iodine and low basal metabolic rate, both suggestive of hypothyroidism [13]. In addition, postmortem analysis of patients who died of heart attacks revealed morphologic changes indicative of thyroid hormone deficiency [14]. Thus, it was not surprising that researchers started considering thyroid hormone therapy as a therapeutic agent in treating CVD.

Enthusiasm for the use of thyroid hormone therapy in CVD was however curbed when it was observed that patients with angina often experienced an increase in symptoms even when physiological doses of thyroid extract were used, especially if therapy was started abruptly. Therefore, it became standard clinical practice to start with low doses and to increase the hormone gradually. It was also believed that synthetic isomer d-thyroxine might be superior to l-thyroxine (the synthetic form of the natural hormone) in decreasing serum cholesterol levels without increasing myocardial oxygen demand [15]. But, a double-blind trial lasting 5 years, the Coronary Drug Project, in patients with a history of myocardial infarction, showed that mortality from the coronary heart disease was similar in the group treated with d-thyroxine compared to the group treated with placebo [16]. It was however noted that the d-thyroxine group experienced remarkably more proarrhythmic effects. This worrying side-effect was probably due to the fact that the dose of d-thyroxine administered (6 mg/day) was equivalent to more than 300 mg of l-thyroxine [17]. In addition, the d-thyroxine preparation used in the trial was contaminated with a high level of active l-thyroxine [18]. Thus, the trial was terminated early and never repeated again.

More recently, data from experimental studies and epidemiological observations have shown that thyroid dysfunction is associated with higher cardiovascular morbidity and mortality [19–21]. Furthermore, even variations of thyroid hormones within the reference range have been linked with adverse events. Therefore, interest in the use of thyroid hormones in the management of CVD has been reawakened.

## Pathophysiology of TH changes in cardiac conditions

The two main thyroid hormones T4 and T3 are secreted by the thyroid gland in response to stimulation by thyroid stimulating hormone (TSH). Both T4 and T3 have biological actions but T3 is considered the active hormone. Thyroid hormone metabolism is regulated by enzymes called deiodinases. Type I (DIO1) and type II iodothyronine deiodinase (DIO2) lead to extrathyroidal T3 production from the precursor T4. DIO1, which is active in the liver and kidney, produces 15–20% of total circulating T3, whereas DIO2 activity located in the pituitary, brain, brown adipose tissue, and heart is responsible for the remaining two-thirds of T3 production [22–24]. DIO3, on the other hand, catabolizes both thyroid hormones and terminates their action. The thyroid hormone status of any given tissue is dependent on both circulating as well as intracellular thyroid hormone levels, which are regulated by deiodinases and thyroid hormone transporters.

**Fig. 1:**
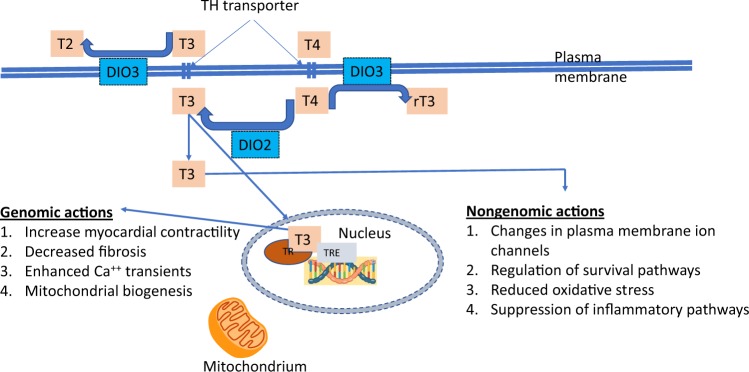
Thyroid hormone actions on the cardiomyocyte Thyroid hormones T4 (thyroxine) and triiodothyronine (T3) enter the cardiomyocyte via plasma membrane transporters. Inside the myocardial cell, T4 can be activated by the enzyme DIO2 (deiodinase 2) to the active form T3, and both T4 and T3 can be inactivated by DIO3 (deiodinase 3) to rT3 (reverse T3) and T2 (diiodothyronine), respectively. From the cytosol T3 diffuses into the nucleus and binds to thyroid hormone receptors (TR) in the presence of TRE (thyroid hormone response elements) and initiates the regulation of target genes and other metabiolic pathways (genomic actions). In addition, thyroid hormones also have direct nongenomic actions. Adapted from Jabbar et al. [3]

Thyroid hormones have wide-ranging effects on the cardiovascular system (see Fig. 1). Thyroid hormones influence cardiac status by several mechanisms: by direct genomic actions through binding to nuclear receptors leading to the regulation of the expression of target genes in the cardiomyocyte; by extranuclear, nongenomic actions on the ion channels located in the cardiomyocyte cell membrane; and through effects of both thyroid hormones on the peripheral circulation [3].

In the nucleus of the cardiomyocyte, T3 binds to thyroid hormone receptors (TR), which in turn bind to thyroid hormone response elements in the regulatory regions of target genes to control transcription. The two main TRs are TRα, which is highly expressed in cardiomyocytes, and TRβ. TRs can bind to thyroid hormone response elements even in the absence of thyroid hormones, leading to repression of transcription of target genes. Thus, gene regulation in the cardiomyocyte is dependent on the availability of thyroid hormones [25]. Thyroid hormones regulate myocardial contractility and systolic function by activation of genes encoding sodium/potassium-transporting ATPases, myosin heavy chain-α, and sarcoplasmic/endoplasmic reticulum calcium ATPase 2, and negatively regulating the transcription of myosin heavy chain-β and phospholamban [3]. Thyroid hormones also have a direct inotropic effect on the heart by positively regulating the gene expression of the β1-adrenergic receptor [26]. In addition, thyroid hormones influence cardiac chronotropy through both genomic and nongenomic effects on components of the adrenergic-receptor complex and on sodium, potassium, and calcium ion channels [27]. The nongenomic effects of thyroid hormones on cardiomyocytes and the vascular system include activation of sodium, potassium, and calcium membrane ion channels, effects on the mitochondrial membrane and mitochondriogenesis, and involvement in signalling pathways of cardiomyocytes and vascular smooth muscle cells [3].

**Fig. 2:**
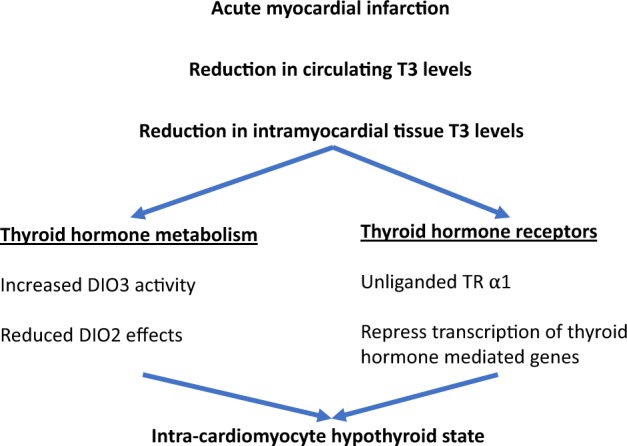
Effects of low serum T3 levels in acute myocardial infarction T3—triiodothyronine, DIO3—deiodinase 3, DIO2—deiodinase 2, TR—thyroid hormone receptor

In cardiac diseases, such as heart failure or acute myocardial infarction, the intracellular cardiomyocyte environment is affected by hypoxia, which in turn leads to inflammation [28]. The hypoxia and the resultant inflammatory response reduce deiodinase activity in the cardiomyocyte, which along with reduced plasma T3 levels, result in a decrease in intracellular T3 bioavailability (see Fig. 2). Furthermore, increased DIO3 gene expression under hypoxic conditions leads to degradation of T3 into inactive metabolites. Therefore, the intracellular environment in the cardiomyocyte in a number of cardiac conditions presents with reduced availability of T3 and, consequently, reduced metabolism. The clinical implications of the reduced bioavailability of thyroid hormones in these patients is unclear.

There are remarkable similarities between developmental processes and changes occurring in the damaged myocardium, which has refocused research on the role of thyroid hormones in the pathophysiology of cardiac disease. Disparate stressful processes such as pressure overload, hypoxia, ischaemia, inflammation, and metabolic disturbances result in the predominance of the foetal gene programming. This genetic developmental switch of the diseased myocardium is not well understood [29]. However, recent evidence suggests that reactivation of the foetal genotype may constitute a permissive state for cell regeneration. There is some evidence that thyroid hormones, which play a critical role during development, may also have an important function in regeneration and repair during adult life [30, 31]. Thus, thyroid hormones may have important therapeutic potential in the management of a number of cardiac conditions.

## Thyroid hormones in heart failure

Thyroid hormones have multiple effects on the cardiovascular system, ranging from molecular and cellular effects to consequent hemodynamic alterations. There is increasing evidence that suggests an adverse relationship between thyroid dysfunction and heart failure (Table 1). In patients with heart failure, both low and high serum TSH levels are associated with higher mortality [32]. Furthermore, higher serum TSH levels are a strong and independent predictor of clinical outcomes including death and cardiac-related hospitalisations [32]. In the SCD-HeFT trial increased serum TSH levels were related to higher mortality in patients with impaired left ventricular systolic function [33]. The other common biochemical abnormality observed in nearly one-third of patients with heart failure is the so-called “Low T3 Syndrome” [34].

The physiological reasons for the development of a low T3 state in patients with severe illness and, in particular, with heart failure, are not clearly understood. There are at least three potential mechanisms that may explain the development of the low circulating T3 levels in patients with moderate and severe heart failure:

1. Reduced activity of DIO1 and DIO2: a decreased extrathyroidal conversion of T4 to T3 due to diminished activity of peripheral deiodinases, associated with a decreased transport of T4 into tissues [35, 36].
2. Increased DIO3 activity: an increase in peripheral thyroid hormone break-down due to an ectopic induction of DIO3 activity in peripheral tissues leading to increased reverse T3 levels I [37].
3. Central hypothyroidism: decreased plasma levels of TSH and by the concomitant abnormalities in thyrotropin releasing hormone [38].

The increased levels inflammatory proteins such as interleukins and cytokines (IL-6, TNF-α, etc.) observed in patients with severe heart failure is associated in both experimental models and clinical scenarios with a pattern similar to low T3 state [39–42]. This observation supports the hypothesis of a link between neuroendocrine and proinflammatory pathways. Moreover, in addition to the above-mentioned changes in hormonal, deiodinase and inflammatory processes, abnormalities in TRs expression in patients with heart failure is also a contributory factor in the development of tissue hypothyroidism [43, 44]. In a rat model of hypothyroidism, T4 replacement therapy normalises plasma thyroid hormone levels, but does not reverse the associated cardiac and vascular changes, suggesting that serum thyroid hormone levels may not accurately reflect tissue thyroid status [45].

The increased cardiac risk in patients with heart failure and low T3 syndrome offers a mechanistic basis for thyroid hormone therapy in patients with left ventricular dysfunction and low/borderline levels of serum T3. In this group of patients, restoration of normal thyroid hormone levels could positively re-establish myocardial gene expression, improve cardiac function and haemodynamics, and thus alleviate morbidity and mortality. A few clinical trials of thyroid hormones have been conducted in patients with heart failure. Moruzzi et al. demonstrated that short- and medium-term levothyroxine therapy in a small number of patients with idiopathic dilated cardiomyopathy (NYHA class II–IV) improved cardiac function and exercise performance, without any significant side effects [46, 47]. In another trial, 23 patients with advanced heart failure (NYHA class III–IV) who were treated with short-term intravenous T3 administration at supra-physiological doses showed an improvement in cardiac output and a reduction in systemic vascular resistance, without significant changes in blood pressure or heart rate [48]. Pingitore et al. randomised 20 patients with heart failure and low T3 levels to treatment with T3 infusion for 3 days at physiological doses or placebo [49]. In this trial, normalising circulating T3 levels were associated with increases in parameters of left ventricular function. In addition, there appeared to be a neuroendocrine reset, with significant decreases in norepinephrine, aldosterone and NT-pro BNP plasma levels [49]. A trial of oral T3 or placebo in 13 patients with stable heart failure and low T3 levels failed to show a significant benefit after 6 months of therapy though [50]. The largest placebo-controlled trial to date of oral T3 in 50 patients with mild to moderate (NYHA class I–III) heart failure and low T3 levels demonstrated benefits in left ventricular function, exercise performance as well as improvement in inflammatory markers [51].

Despite these results, the small number of patients studied, the relatively short duration of treatment, and differences in the route and dose of T3 used, limit interpretation of results. Nonetheless, these results suggest that T3 therapy in patients with stable chronic heart failure may be beneficial. Larger multicentre trials for longer periods that also provide information on hard clinical outcomes such as mortality, arrhythmias, and hospitalisations are required before thyroid hormone therapy can be routinely prescribed in patients with heart failure as part of clinical practice.

## Thyroid hormones in acute myocardial infarction

Thyroid hormone action is important for myocardial contractility, acts as a vasodilator and has a direct positive action on myocardial mitochondrial function. Furthermore, antiaptotic and antifibrotic effects of thyroid hormones may also attenuate maladaptive cardiac remodelling and improve myocardial function post-AMI. Reduction in concentration of the active thyroid hormone T3 in ischaemic cardiomyocytes has also been linked to the pathogenesis of ischaemia-reperfusion injury [52]. In rodent models of AMI, a state of tissue hypothyroidism is observed, independently of circulating T3 levels [53]. In AMI patients undergoing reperfusion therapy, subsequent reduction in circulating T3 levels represents a strong independent predictor of death and major adverse cardiac events [54]. In addition, subclinical hypothyroidism in cardiac patients is associated with poor prognosis [55]. Therefore, thyroid hormone therapy in AMI patients may offer a potentially useful option to limit ischaemia-reperfusion injury and reduce infarct size, and also improve postinfarct left ventricular function as well as morbidity and mortality.

### Data from experimental studies

Thyroid hormones have several effects on the cardiovascular system that are relevant to the pathophysiology of the injured cardiomyocyte after an AMI. T3 plays a critical role in regulating mitochondrial function and morphology, modulating antifibrotic and proangiogenic effect, and also by impacting on cell regeneration and repair [3]. Numerous studies in animal models of AMI have shown that thyroid hormone treatment commenced a few hours to a week after AMI limits ischaemia-reperfusion injury and increases post-ischaemic myocardial functional recovery [56, 57]. This effect seems to be of therapeutic relevance for supporting haemodynamics in the clinical setting of ischaemia-reperfusion, such as myocardial infarction or cardiac surgical procedures.

Thyroid hormones are essential in the regulation of molecular mechanisms of angiogenesis, cardioprotection, cardiac metabolism, and myocyte regeneration—processes that can modulate recovery from injury and left ventricular remodelling. Based on experimental studies, the protective effect of thyroid hormone on reducing ischaemic reperfusion injury and improving cardiomyocyte remodelling and function is considered to be mediated by:

- Antiapoptotic and mitochondrial protection by modulating mitochondrial permeability transition pore (MPTP) opening [58–60].
- Positive effects on the thyroid hormone deactivating enzyme DIO3, MCT-10 (thyroid hormone transporter) and foetal gene re-expression, and thus restoring cardiomyocyte T3 levels [28].
- Attenuates infarct fibrotic area and reverses foetal gene expression [61].
- Improved myocyte preservation in the peri-infarct zone [62].
- Facilitate recovery of stunned myocardium and left ventricular function with positive effects on alpha- and beta-myosin heavy chain (MHC) and SERCA/Phospholamban ratios [63, 64].

### Data from clinical observations and trials

There have been a number of studies in patients with AMI that have evaluated T3 levels with morbidity and mortality [65]. Most studies have reported an adverse association between low circulating T3 and all-cause mortality, troponin levels, myocardial area at risk, or cardiovascular complications. These observations, however, cannot ascertain a causal association between the two conditions, and, therefore, interventional trials of T3 are required to investigate the link.

There is little data on the safety and efficacy of T3 in AMI patients apart from a recent phase II trial [66]. In this randomised controlled trial of oral T3 in 37 patients with STEMI and low serum FT3 levels, T3 replacement therapy for 6 months was shown to be safe and effective in reducing regional cardiac dysfunction. Left ventricular ejection fraction and myocardial infarct size, as assessed by cardiac MRI, did not show any benefit. In this trial, T3 therapy was commenced 72 h after the onset of STEMI, and the preparation was administered three times per day. This small trial provides reassurance that normalisation of serum T3 levels in patients with STEMI who have low circulating T3 levels is safe, if T3 therapy is comenced a few days after the acute event. Another trial of levothyroxine in AMI patients with subclinical hypothyroidism is ongoing with the aim to evaluate effects on left ventricular function [67]. However, trials of thyroid hormones in AMI in larger groups of patients and treated for longer duration are required to confirm safety and efficacy before thyroid hormone supplementation can be considered for routine use in these patients.

## Thyroid hormones in cardiac surgery and cardiac transplantation

There are several trials of thyroid hormones, particularly T3, in other cardiac conditions that provide useful information and help to draw valuable conclusions. T3 administration in patients undergoing cardiac surgery such as coronary artery bypass grafting, a procedure in which myocardial tissue is subjected to ischaemia-reperfusion injury, reduces troponin release and improves post-surgery haemodynamics [68]. Similarly, thyroid hormone administration in children undergoing surgery for congenital heart defects protects against myocardial ischaemia-reperfusion injury [69]. Subgroup analysis of a large randomised clinical trial (TRICC) showed a significant reduction in extubation time and use of inotropic drugs, and better cardiac function with T3 supplementation after surgery in paediatric patients aged <5 months [70].

Thyroid hormone use can have important implications for organ selection and cardiac function before and after transplantation. Experimental studies demonstrated a surge of adrenaline levels after the induction of brain death in a baboon, which was associated with rapid declines in plasma levels of various hormones including T4 and T3 [71]. The reduction in thyroid hormone levels after brain death can lead to significant impairment of cardiovascular function partly due to impairment in aerobic respiration [72, 73]. Hormonal therapy that included T3 in a series of brain-dead potential organ donors resulted in improved outcomes. A retrospective analysis of more than 66,000 brain-dead potential organ donors in the USA revelaed that thyroid hormone therapy increased in the number of transplantable organs by nearly 13% [73]. A survey of 24 organ procurement organisations in the USA showed that more than 70% utilise thyroid hormones in all potential donors although there is large variation in the dose used [74]. A consensus statement by opinion leaders recommended the use of hormonal resuscitation, including T3, to maximise use of potential organs [75].

## Conclusion

Current experimental and clinical evidence suggests that thyroid hormone treatment may be beneficial in improving cardiac function and that risks of arrhythmias appear to be low. However, despite encouraging indications from experimental studies, large adequately-powered interventional trials of thyroid hormones in patients with various cardiac conditions have not been conducted. One explanation for this is the common belief of physicians that exogenous thyroid hormone (or its analogues) therapy in cardiac patients could precipitate development of arrhythmia, myocardial ischaemia/ infarction, or worsening of congestive heart failure.
